# Autonomous vehicles are cost-effective when used as taxis

**DOI:** 10.1186/s40621-018-0153-z

**Published:** 2018-06-04

**Authors:** Isaac G. Freedman, Ellen Kim, Peter A. Muennig

**Affiliations:** 1 Yale School of Medicine, New Haven, USA; 2 Global Research Analytics for Population Health, Columbia University Mailman School of Public Health, New York City, USA; 3 University of Michigan School of Public Health, Ann Arbor, USA

## Abstract

**Background:**

Autonomous vehicles (AVs) will radically re-shape the health and well-being of people in the United States in good ways and bad. We set out to estimate a reasonable time-to-adoption using cost-effectivenessmodels to estimate the point at which AVs become reasonably safe and affordable for widespread adoption.

**Methods:**

We used Waymo data (previously, Google Self-Driving Car Project) and a microsimulation model to explore projected costs and safety issues today and five years from today to get a sense of the speed of consumer adoption were AVs brought to the market.

**Results:**

The adoption of AVs for private use was associated with an ICER of 1,396,110/QALY gained today, a figure that would decline to 173,890/QALY gained 5-years in the future. However, AV taxis are both less expensive and potentially already safer than human-piloted taxis.

**Conclusions:**

While AVs are not unlikely to be used a family vehicles any time soon, it would make economic sense to adopt them as taxis today. Legislation enhancing the benefits while mitigating the potential harmful health impacts of AV taxis is needed with some urgency.

**Electronic supplementary material:**

The online version of this article (10.1186/s40621-018-0153-z) contains supplementary material, which is available to authorized users.

## Background

Autonomous Vehicles (AVs) are presently on the road with no human behind the wheel in some localities (Energy and Commerce Committee 2017; U.S. Department of Transportation 2016; State of California Department of Motor Vehicles 2018). These “level 4” vehicles are confined to certain conditions and roads, but could plausibly be introduced more broadly in the coming year. The Self Drive Act (HR3388) is designed to override and unify state regulations, with the ultimate goal of expediting the transition to a future without human piloted vehicles (HPVs). Federal lawmakers so far are nearly exclusively focused on short-term issues, such as safety, licensing, and insurance issues (Energy and Commerce Committee 2017).

However, the potential benefits and harms of AVs extend beyond their immediate safety on the road or reduced insurance costs. AVs have the potential to transform our lives in ways unseen since the introduction of automobiles themselves well over 100 years ago. Without policies to foster their benefits and mitigate their harms, it is at least conceivable that society could end up with a public health threat equal in magnitude to the one posed by the introduction of private cars over a century ago. At that time, policymakers did not foresee the automobile as a leading cause of death globally. Rather, they focused on licensure and ownership, overlooking its potential impact on injuries, fatalities, a sedentary lifestyle, obesity, and air pollution (McGinnis and Foege 1993).

While some level 4 AVs have been mixed with HPVs, they are not presently on the market for sale (Kang 2017). We undertook this analysis because cohesive, well-informed legislation is needed that considers both the short-term public health benefits and the longer-term social consequences of AVs (Business Insider Insider Reports 2015; KPMG 2013). The longer-term consequences will require time to study and regulate. We do not evaluate these potential long-term challenges. Rather, in this study, we simply ask whether it is plausible that AVs are affordable and safe in their current state today and five years from today. If AVs are presently unaffordable or are dangerous, they are unlikely to be widely adopted by consumers irrespective of whether laws permit their use. Under these circumstances, governments may debate and study the unintended consequences of introducing driverless cars on our roads. As the technology improves and becomes cheaper, laws can be gradually put into place that mitigate the harms of AVs while nurturing their benefits.

However, if AVs are currently both affordable and reasonably safe relative to conventional HPVs, the government should rapidly make large investments in understanding the benefits and threats posed by AVs. The urgency arises from two competing problems that arise when this new, affordable, and safer technology is available. Either: 1) society will fail to deploy AV technology with the potential to save lives in the short-term, leading to needless suffering and loss of life; or, 2) AVs will be successfully introduced on the road over broader geographic areas and will continue to grow in use with little regulation, leading to potentially serious long-term health and social consequences.

In this study, we simulate the economic costs and health benefits of AVs today given their high costs as well as the considerable uncertainty surrounding their safety. We simply ask whether AVs are affordable and plausibly safe given the best available data. We also make projections of future costs.

## Methods

### Analytic overview

While AVs frequently hand control over to a human driver and perform poorly in bad weather (known as disengagement) (State of California 2015; Favaro et al. 2018), there is now sufficient data to estimate crash rates were no driver present when software is optimized for a given city.

Waymo (previously, Google Self-Driving Car Project) uses a wider array of expensive technology to improve its safety than other commercial AVs, and Waymo’s vehicles have logged over 2 million miles, with billions of simulated miles (Waymo 2017). These data allow for microsimulation modeling of future crashes, costs, and health effects. We further consider multiple alternative scenarios of the unintended consequences of AVs.

We conducted a model-based analysis that closely modeled the publicly-reported results from Waymo since December 2016, the last point at which publicly-available data were posted online (Waymo 2016b). The dataset included a list of 14 crashes that took place between 1 October 2012 and 22 August 2016 while completing 2,102,047 miles of fully autonomous travel. While the car was on fully-autonomous mode for all crashes used in our analysis, an engineer was in the car at all times. The average speed of the AV during collisions was 0.23 miles per hour and the average speed of the other vehicle in collisions was 7.54 miles per hour, with the majority of crashes (9 of 14, 64%) being rear-end collisions with the AV stationary. We used a Markov chain microsimulation model that compares AVs and HPVs over the average lifespan of a car. All future costs and QALYs were discounted at a rate of 3%, and we adhered to the reference case scenario of the new Panel on Cost-Effectiveness Analysis for Health and Medicine (Neumann et al. 2016). Our chief assumptions are listed in Table 1.

**Table 1:** Major assumptions and their justifications used to build our Markov microsimulation models

Assumption	Justification
Driverless autonomous vehicles (AVs) will have a higher minor crash rate than human piloted vehicles	Minor crash rates of human-piloted vehicles are very difficult to estimate as most go unreported. Simulations from Waymo show lower crash rates, but the company has a financial stake in such outcomes. Reported minor crash rates for Waymo showed higher rates in AVs as compared to HPVs. The Waymo dataset is a key data source for this study.
2 million miles of driving is adequate for inference regarding the safety of AVs	We used established formulas to extrapolate probabilities of injury and death using observed mean crash speeds for AVs.
If adopted today, AVs would eliminate most parking spaces	AVs can be rented as taxis by private car owners when not being used.
The cost of autonomous vehicles will decline following Moore’s law	The efficiency and cost of many technologies follows Moore’s law for central processing unit speeds.
Used cars will have a similar market, whether human piloted or autonomous	While technologies in AV have few moving parts, they tend to decline in value as fast as automobiles do because the technology becomes dated very quickly.
Autonomous vehicles without a human driver will increase productivity	When the driver’s seat can hold a paying passenger, the additional passenger will sometimes perform work on a device (e.g., emailing colleagues).
AVs and human-piloted vehicles are of similar quality of build	The build of a car can also influence the probability of injury and death. In current use, AV equipment seems to be used across a span of makes and models.

Using a representative cohort of drivers with ages reflecting the ages of US drivers, we calculated the incremental cost-effectiveness ratio (ICER) of AVs as they exist today relative to HPVs, while also projecting various future expected and possibly unintended consequences of adopting AVs. Specifically, we estimated the incremental change in cost divided by the incremental change in QALYs of AVs relative to that of HPVs, accounting for the differential in QALYs accrued when injured or dead, and various costs.

### Costs

We calculated the total costs of property damage only, minor, and severe injury crashes as classified by the Maximum Abbreviated Injury Scale (MAIS) score, and death using data obtained from the National Highway Traffic Safety Administration (NHTSA) report on the economic and societal impact of motor vehicle crashes (Blincoe et al. 2015). We considered a MAIS score of less than 3 to be a minor injury crash and a MAIS score of 3 or above to be a severe injury crash. These HPV crash costs were then scaled to more accurately reflect AV crashes since AV equipment is more expensive (Greenblatt and Saxena 2015; Vallet 2016). The scaling of costs was achieved by estimating the proportion of costs attributable to destruction of a vehicle (car damage) and adding the expected marginal cost of damage to an AV relative to a HPV in a similar crash type (see Additional file 1: Equation 2). We estimated the average costs of a HPV (New-car transaction prices up 2 percent in march 2016, along with increases in incentive spend, according to Kelley blue book [press release] 2016), an AV (Greenblatt and Saxena 2015), an AV projected five years into the future under the assumption that the cost of technology follows Moore’s law—i.e., periodic halving—with a period of 2 years (Greenblatt and Saxena 2015; Strawn and Strawn 2015), the annual cost of owning a car (American Automobile Association 2014), average cost of a funeral (National Funeral Directors Association 2017), productivity loss (i.e. opportunity cost) from driving (United States Office of Personnel Management n.d.; American Automobile Association 2015; Proctor et al. 2016), costs of spots (The Economist 2017; Chester et al. 2010), and taxi-related costs (Greenblatt and Saxena 2015; New-car transaction prices up 2 percent in march 2016, along with increases in incentive spend, according to Kelley blue book [press release] 2016; Bureau of Labor Statistics 2015; New York City Taxi and Limousine Commission 2014) using various other sources and assuming national averages. Productivity loss from driving a HPV was calculated by multiplying the mean wage (United States Office of Personnel Management n.d.; Proctor et al. 2016), mean commuting time (American Automobile Association 2015), and a productivity parameter between 0 and 1, with a base case productivity parameter of 0.3 Cost of parking spots for non-taxis were calculated by multiplying the average cost of one parking spot (including costs to build, buy the land, maintain the spot, etc.) (The Economist 2017) by the ratio of parking spots to registered cars (base case ratio of 3.4) (Chester et al. 2010). The cost of parking spots for taxis were set equal to just the average cost one parking spot (The Economist 2017). Taxi-related costs included costs for buying and maintaining the vehicle, as well as salaries for drivers. We assumed that increased parking availability, as a result of taxi usage, would reduce fuel costs and offset the additional fuel costs from using vehicles as taxis. See Additional file 1 for more notes on cost of taxis. All parameters used in estimation were included in sensitivity analyses. All costs were inflated to 2016 US dollars and are shown in Table 2.

**Table 2:** Selected costs and probabilities used in the Markov microsimulation model

	Low Value	Base Value	High Value
Selected Costs ($)^a^			
Property Damage Only Crash (Blincoe et al. 2015)	2976	4251	5526
Minor Injury Crash (Blincoe et al. 2015)	3129	12,282	61,352
Severe Injury Crash (Blincoe et al. 2015)	200,241	274,553	1,101,865
Fatal Crash (Blincoe et al. 2015)	1,072,412	1,532,018	1,991,623
Property Damage Only Crash AV (Blincoe et al. 2015; Greenblatt and Saxena 2015; Vallet 2016)	3123	4461	5799
Minor Injury Crash AV (Blincoe et al. 2015; Greenblatt and Saxena 2015; Vallet 2016)	3864	13,332	62,717
Severe Injury Crash AV (Blincoe et al. 2015; Greenblatt and Saxena 2015; Vallet 2016)	214,206	294,503	1,127,800
Fatal Crash AV (Blincoe et al. 2015; Greenblatt and Saxena 2015; Vallet 2016)	1,086,965	1,552,808	2,018,650
Autonomous Vehicle (Greenblatt and Saxena 2015)	122,218	183,666	265,648
Autonomous Vehicle in 5 Years (Greenblatt and Saxena 2015; Strawn and Strawn 2015)	33,229	56,539	100,383
Human Piloted Vehicle (New-car transaction prices up 2 percent in march 2016, along with increases in incentive spend, according to Kelley blue book [press release] 2016)	17,218	33,666	70,648
Annual Cost of Owning Car (American Automobile Association 2014)		8536	
Funeral (National Funeral Directors Association 2017)	6206	7332	8687
Productivity (United States Office of Personnel Management n.d.; American Automobile Association 2015; Proctor et al. 2016)	552	1657	2762
Parking spot for non-taxi (The Economist 2017; Chester et al. 2010)	47,600	102,000	176,800
Parking spot for taxi (The Economist 2017; Chester et al. 2010)	0	30,000	80,000
Taxi Salary (Bureau of Labor Statistics 2015)	19,432	27,760	36,088
Cost of Taxi (New-car transaction prices up 2 percent in march 2016, along with increases in incentive spend, according to Kelley blue book [press release] 2016; New York City Taxi and Limousine Commission 2014)	92,699	132,427	172,155
Cost of AV Taxi (Greenblatt and Saxena 2015; New York City Taxi and Limousine Commission 2014)	197,699	282,427	367,155
Selected Probabilities			
Human Piloted Vehicle (Blincoe et al. 2015; National Highway Traffic Safety Administration 2016)			
Crash	–	0.0646	–
Property Damage Only Crash	–	0.6091	–
Minor Injury from Crash	–	0.3766	–
Severe Injury from Crash	–	0.0090	–
Death from Crash	–	0.0054	–
Autonomous Vehicle (Waymo 2016b)			
Crash	0.06729	0.08385	0.09319
Property Damage Only Crash	0.9551	0.9654	0.9777
Minor Injury from Crash	0.0220	0.0315	0.0409
Severe Injury from Crash	2.09E-04	2.99E-03	3.88E-03
Death from Crash	6.48E-05	9.26E-05	1.20E-04

^a^All costs have been rounded to the nearest 2016 dollar

### Probability of crash, injury, and death

Expected annual probabilities of injuries and fatalities in HPVs were calculated via NHTSA reports (Blincoe et al. 2015; National Highway Traffic Safety Administration 2016). We use data from 2,102,047 autonomous vehicle miles travelled (VMT) collected between 2010 and 2016 from Waymo – a period over which there were 14 crashes (Waymo 2016b). In all cases, the driver of the non-AV was at fault. While all Waymo crashes are reported, many HPV crashes are not. When only reported crashes are considered, AVs are 1.30 times more likely to be involved in a minor crash (Table 2).

Waymo’s AVs are tested with a human behind the wheel who can take over if needed. Waymo’s simulations predict that there would have been an additional 7 crashes between 2010 and 2016 were the human not able to take control of the car, and that all 7, or 1/3, would have been caused by the AV. Given the small *n* of 21 crashes, we chose not to use actual crashes to estimate injuries and fatalities. Rather, we used well-established formulas that outline the risk of crashes, injuries, and fatalities based on mean change in vehicle speeds at the time of the crash (Mohit et al. 2017; Flannagan 2013), whether caused by the AV or the human driver of the other car. Using the variance of the actual distribution of speeds at the time of crash, we created a distribution of speeds at the time of the crash, and linked each sample of this distribution to its risk of injury or death.

The mean change in speed (∆V) for Waymo crashes was 3.91 miles per hour. This injury model classified injuries using the KABCO scale in which an individual is either killed (K), has an incapacitating injury (A), a non-incapacitating injury (B), a possible injury (C), or no injury (O). In order to use data reported in both MAIS and KABCO format, we considered the two scales equivalent, with K corresponding to MAIS 6, A corresponding to MAIS 3, 4, or 5, B corresponding to MAIS 1 or 2, and C corresponding to MAIS 0 or no injury.

### Morbidity

Changes in health-related quality of life due to MVCs were derived using the EuroQol 5D5L with the assistance of two pediatric orthopedic surgeons with extensive clinical experience working with crash victims (Muennig et al. 2014; Gold et al. 1996).

### Decision-analysis models

We simulated the average lifespan of a motor vehicle with an average of 14,133 annual VMT per licensed driver (National Highway Traffic Safety Administration 2016). A decision-analysis model was constructed using TreeAgePro 2016 software for the Macintosh computer (TreeAge Software, Willamstown, Mass.). The model examined the use of a mode of transportation for the lifespan of a HPV compared to the use of an alternative mode of transportation for the same time period.

Following is a description of the baseline HPV versus AV model. For each iteration (*N* = 10,000) of the decision analysis model, two drivers were simulated, each with the same age randomly selected from a representative distribution of licensed drivers’ ages in the United States. The driver was randomized to either pilot a HPV or AV. At each time step for either vehicle, the subjects could be in one of several states: driving, driving severely injured, dead, out of the analytic horizon and healthy, or out of the analytic horizon and severely injured. The analytic horizon was defined as the lifespan of a representative vehicle, taken from a vehicle lifetable, and an AV was assumed to have an equivalent lifespan to that of an HPV (Table 1). It was important to continue to run the model after the analytic horizon was reached, so as to accurately calculate the impact on total QALYs of each strategy past the lifespan of the car. While driving or driving severely injured, a subject accrued the costs of purchasing a vehicle (only on the first timestep), the cost of owning and operating a vehicle, the cost of parking, and the productivity loss from piloting a vehicle ($0 for AV) (Table 2). For models involving taxis, the salary of the taxi driver and other costs were included. Both the expiration of the car (i.e., the end of the analytic horizon) and the probability of a crash were drawn from uniform random variables, as in a Monte Carlo model, and compared to a lifetable for each timestep. The cost of vehicle maintenance and upkeep for an AV was equal to the cost of maintenance for a HPV plus the relative marginal cost of an AV relative to an HPV—i.e., the cost was scaled to account for the relative difference in vehicle cost. For each timestep, a subject could be involved in a crash with a fixed probability. The crash type was determined using national statistics of the frequency of crashes of each MAIS type, where an of MAIS 0, 1, and 2 was considered a minor injury crash, an MAIS of 3, 4, or 5 was considered a severe injury crash, and a fatal crash and property damage only crash were considered in their own crash categories. If an individual was involved in a fatal crash, they would be sent to the “Dead” category for subsequent time steps, while if an individual was involved in a severe injury crash, they would be sent to the “Driving Severely Injured” category. Individuals could also be sent to the “Dead” category based on a lifetable checked each year given the subject’s age. At the conclusion of the model (*T* = 100 timesteps), all vehicles were expired and all individuals were dead; the QALYs and costs for each iteration (*N* = 10,000) were calculated and compared within iterations and across the entire simulation.

The decision trees for each model can be found in the Additional file 1.

### Sensitivity and scenario analyses

We first conducted an analysis for the base-case scenario, defined by the input values in Table 2. Sensitivity analyses were then conducted using plausible ranges of high and low values for each variable to test their influence on the results of the model using the range of values in Table 2. Values for several variables of interest were simultaneously varied over their plausible range using Monte Carlo microsimulations (*N* = 10,000 microsimulations), with values drawn from probabilistically weighted triangular distributions or from reported heterogeneous distributions, with linear interpolation. Because NHTSA does not publish a standard willingness-to-pay (WTP) threshold, we used the EPA’s WTP threshold of $140,000/QALY (Muennig and Bounthavong 2016). The results of this threshold analysis are reported below.

## Results

### Simulation of human-piloted car cohort

The model replicated the U.S. cohort of conventional car drivers between 2013 and 2016 and the U.S. cohort of conventional taxis. Over the lifetime of the average car, HPVs cost about $286,000 including the cost of the vehicle, maintenance, parking, and other costs. The average number of QALYs that the driver lives is 16.43.

### Projected outcomes for the adoption of autonomous vehicles

#### Quality-adjusted life years

Data from Waymo indicate the relative risk (RR) of crashes for AVs is 1.30, while the RR for fatalities based upon the vehicle’s speed at the time of the crash is 0.02 (Table 1) due to a much lower speed of impact with any collisions.

AVs were projected to be safer than HPVs, with an incremental increase in QALYs of 0.08 (0.05% change), or approximately one month of perfect health, over the time that the car is on the road.

#### Costs

The vehicle lifetime costs of purchasing, maintaining, and operating new AVs ($425,757, 95% CI: $288,479–$594,010) were projected to exceed costs for HPVs over the same period by 49%, with most of the costs attributed to the initial cost of an AV. The lifetime costs of purchasing, maintaining, and operating a new AV with costs reduced by 5-year Moore’s Law projection ($303,535, 95% CI: $173,959–$613,812) still exceed the costs for HPVs by 6%. The projected lifetime cost of using AV taxis ($447,667, 95% CI: $306,002–$634,870) in place of HPVs was found to be 56% more expensive than owning and operating a private HPV for the same period. The difference in costs is primarily due to profits for the taxi owner. The projected lifetime societal cost of using a human-piloted taxi ($570,032, 95% CI: $222,787–$1,205,646) was 34% higher than those for using an AV taxi over the same period. Since both vehicles have similar parking requirements, the difference was primarily due to the salary of the human driver.

#### Cost-effectiveness

The adoption of AVs for private use was associated with an ICER of $1,396,110/QALY gained (Table 3). Likewise, the projected adoption of AVs in 5-years was $173,890/QALY gained. The scenario comparing current HPVs to AV taxis was associated with an ICER of $1,615,210/QALY gained. The strategy of using human-piloted taxis as an alternative to AVs was found to save both money and QALYs.

**Table 3:** The cost, quality-adjusted life years gained, and 95% confidence intervals (CIs), and incremental cost-effectiveness ratios (ICER) from our microsimulation models (based on 10,000 microsumulations) for human piloted vehicles (HPVs) versus: privately-owned autonomous vehicles (AVs), AVs 5 years in the future, and AV taxis. The final simulation compares human piloted taxis versus AV taxiss

	Cost	95% CI		QALYs	95% CI		ICER
		2.50%	97.50%		2.50%	97.50%	
Human-Piloted Vehicles Versus AVs							
HPVs	286,146	155,949	653,505	16.41	0.99	28.18	
AVs	425,757	288,479	594,010	16.51	1.47	28.34	1,396,110
AVs in 5 Years^a^	303,535	173,959	613,182	16.51	1.47	28.34	173,890
AV Taxis	447,667	306,002	634,870	16.51	1.47	28.34	1,615,210
Human-Piloted Taxis Versus AV Taxis							
HPT	570,032	222,787	1,205,646	16.41	0.99	28.18	
AV Taxis	447,667	306,002	634,870	16.51	1.47	28.34	Saves money and QALYs

Incremental cost-effectiveness ratios in this analysis are only useful insofar as they inform consumers of the value of an AV relative to a conventional car on the grounds of the best available health data. These data are subject to considerable uncertainty

^a^AVs were assumed to fall in price according to Moore’s Law, but were not assigned increased effectiveness

Cost-effectiveness estimates were remarkably stable for all scenarios and variables, with two notable exceptions. The model comparing HPVs to a 5-year projection of AVs was sensitive both to variation in the cost of an AV or AV taxi and to variation in the probability of crash in an AV or AV taxi.

## Discussion

While the data are poor, and there is considerable uncertainty, we find that the widespread adoption of AVs could plausibly save lives, reduce suffering, and produce improvements in productivity (due to fewer injuries), in the short-term. However, it is unlikely that such vehicles will find much of a market for personal use in private sector in the near-term due to their high cost of ownership and the impracticality of limiting the vehicles to roads on which they have been programmed to operate. This is true even when additional cost savings are considered, such as the economic benefits associated with reductions in parking spaces.

However, we find that AVs would save money if used as a taxi, even in their current state of development. Both companies and families might be incentivized to invest in an AV were one available on the market. Such a vehicle could be rented out for additional income as a ride share, rather than depreciating in value in the garage. This could effectively incentivize fairly widespread adoption of this technology, dramatically reduce the cost of ride sharing, and remove jobs. For this reason, legislators should consider studying the regulation of AVs with more urgency. If a commercially viable product ends up on the road, the widespread adoption of AVs could come sooner than anticipated even if its market price is too high for most families to afford as a personal vehicle.

For this reason, policies that mitigate the potential harms of AVs—and particularly the widespread and rapid use of AVs as taxis, should be studied with more urgency.

Many have speculated about the potential harms and benefits of AVs on the road. If adopted today, AV taxis would likely make it easier to transport people with minor disabilities and would also lower costs for taxi trips. This way, AV taxis produce large benefits for the small segment of the population that regularly relies on taxis or disabled transit services. However, it could also expand the number of people who opt to ride share rather than own a personal vehicle.

When used as taxis, AVs could, in the long-term, also plausibly produce more land in cities for real estate, sidewalks, bike lanes, emergency vehicle lanes, and parks. This is because they continuously drive, shuttling people around, and rarely needing to park. By decreasing cars owned for personal use, they could eventually eliminate office and shopping mall parking spaces, as well as parking spaces provided by cities. By reducing congestion and increasing drop off space, AVs could reduce transport times of goods, increasing economic efficiency at the macro level. Driver costs consume upwards of 30% of trucking costs (European Parlament 2015).

While this brings huge economic efficiencies for corporations, it can produce large shifts in the labor force. These include the rapid displacement of taxi and delivery drivers who might not be able to find jobs that pay as well. By providing taxi services at a lower price, AV taxis could also move middle-income riders out of urban public transportation systems, which too often function on the edge of financial viability in the US. (Many countries tend to have cheaper, more stable transit systems, however.)

Because they deliver passengers directly from one destination to the next and because they displace public transit, AV taxis could lead to less walking and higher rates of obesity among some segments of the population, while incentivizing others to take to the safer roads for exercise.

Any of the above scenarios are mere speculation. Without investments in research to study these potential impacts of AVs, including regulatory policy experiments, there will be no public debate or preparation for these possibilities.

If the time horizon for a shift to AVs is a year to a few years, as our study suggests it might be, action is needed sooner rather than later. It is certainly difficult to study whether AV taxis would reduce congestion and pollution (by driving very close to one another and by optimizing routes) or would increase it (by increasing demand for road vehicles). However, legislation is best made on research rather than guesswork, and complex systems dynamics models could inform such policymaking (Sterman 2006).

We also studied the cost-effectiveness of AV technology in as a health investment because it is possible that some families would be attracted to the safety of AVs relative to conventional vehicles. At present, AV adoption in passenger vehicles is significantly more expensive per QALY gained than medical practices that are deemed unaffordable (Boulware et al. 2003). It would be more than 5 years before privately owned AVs reached the $140,000/QALY gained threshold, which is considered a high valuation for the willingness-to-pay for a QALY (Muennig and Bounthavong 2016). As such, they are not a good value even for households that can afford them for personal use, nor are they likely to be in the near future.

### Limitations

Our study has several limitations ranging from data uncertainty in present terms to wide-ranging assumptions about what the future might look like. We tested this uncertainty and these assumptions using multiple one-way sensitivity analyses and a Monte Carlo analysis, which examines all sources of uncertainty together.

One of the biggest challenges in building our model was estimating crash rates. Roughly, 47% of crashes among HPVs go unreported (M. Davis and Co. 2015). This is most likely because it is often less expensive for owners to directly pay for minor automobile damages than to make an insurance claim. On the other hand, our AV data contain every incident, no matter how minor the crash. As a result, it is difficult to know whether AVs actually cause more or fewer minor crashes.

Waymo’s simulations based on billions of miles of simulated driving predict that there would be only 68% as many minor crashes if AVs completely replaced HPVs today (and many fewer serious ones). We chose not to rely on Waymo’s simulation data to estimate minor crash rates because the data are not available for public scrutiny. But if we did use this estimate, the projected safety of AVs would be greatly increased, along with their cost-effectiveness.

Second, the non-simulated data from Waymo that we do use not only depends on reporting by the company itself, but are also limited to just over 2 million miles of actual road driving.

Finally, AV crashes may garner more attention than HPV crashes (Boudette 2016). If adopted too early, the crashes that they do cause may stir backlash, derailing their use. Our models simply show that AVs could plausibly be used on the road today under ideal conditions.

One of the more obvious threats is that, with AV taxis on the road, society could lose public transit systems. If so, they pose threats to mobility for low-income Americans, and those in major urban areas that are dependent on public transit systems.

## Conclusions

In the early 1900s, horse manure and corpses littered the streets of major cities, posing a public health threat. When the automobile was first introduced on US streets, it was seen as a way of mitigating these public health threats, but little attention was paid to the public health threats that automobiles produced as unintended consequences. We are now at the next frontier of transportation. However, once again, Federal legislation is focusing on mitigating the public health threats associated with human-powered vehicles without attending to the threats that they pose. Our study shows that autonomous taxis appear to be on the threshold of viability from an economic and injury prevention standpoint and may be introduced more rapidly than some experts believe. There is an urgent need for legislators to begin to focus on the unintended consequences of AVs, including their impact on public transportation and the built environment.

## Additional file

Additional file 1: Supplemental Appendix including notes on calculations; complete lists of variables, life tables, and decision trees; and example results. (DOCX 12099 kb)
