# Supplementary material for: Autonomous vehicles are cost-effective when used as taxis
Source: Inj Epidemiol. 2018 Jun 4;5:24. doi: 10.1186/s40621-018-0153-z (PMC5985243; doi:10.1186/s40621-018-0153-z)
Supplement: Supplementary file 1 — Supplemental Appendix including notes on calculations; complete lists of variables, life tables, and decision trees; and example results. (DOCX 12099 kb) [file 40621_2018_153_MOESM1_ESM.docx]

**Additional file 1**

**NOTES ON FATALITY RATES**

The fatality rate in the US is roughly 1 per 100 million miles driven. It is therefore impossible to directly compute fatality rates given that Google AVs have logged and published data on only 2.3 million miles at the time of writing. However, Google has simulated 6.5 billion of miles of driving.

Tesla has two reported fatalities in semi-autonomous mode with 1.3 billion miles driven, or roughly 0.002 fatalities per HPV. However, there could be many other deaths that went unreported and AVs were assisted by human drivers in all instances.

**NOTES ON COST OF TAXIS**

To calculate the net present cost of purchasing a taxi, we compared the rate of replacement for taxis versus the rate of replacement (lifespan) of personal vehicles.

In New York City, taxicabs drive an estimated 70,000 miles per year, 5-times the annual miles driven by an average non-taxi personal vehicle. We took this 5-times increase as an extreme value for a highly populated urban center and estimated that taxis are replaced at approximately 3-times the rate of personal vehicles.

The net present cost (NPC) of a taxi was estimated as the initial purchase price of an average taxi vehicle, plus the future discounted purchase price of purchasing that vehicle multiple times in subsequent years up until the average lifespan of a personal vehicle. This is represented by the equation

$$NPC\left( T,r \right)=\sum_{Nt=0}^{Nt<T} Disc\left( C_{0}, Nt,r \right)$$

**Supplemental Equation (S.E.) 1.**

Where, C_0_ is the current cost of a taxi vehicle, r is the discount rate (here, r = 0.03), Disc(.) is the discounting function, and N is the relative replacement rate (e.g., 3 for 3-times as frequently replaced). For an average care lifespan of T = 11 years with N = 3, S.E. 1 becomes

$$NPC\left( 11 years, 0.03 \right)=C_{0}+\frac{C_{0}}{{(1+r)}^{3}}+\frac{C_{0}}{{(1+r)}^{6}}+\frac{C_{0}}{{(1+r)}^{9}}$$

We used this formula to calculate the costs of all taxis.

**NOTES ON COSTS OF AV CRASHES**

The cost of an AV crash must include the additional cost of potential damage to the vehicle itself. In our models, AVs are more expensive than HPVs, so the cost of damaging an AV should be higher in proportion to the relative marginal cost of an AV. Additionally, the increased cost of an accident in an AV should have a greater effect for more severe accidents—i.e., accidents where collateral damage is more likely.

The cost of a given type of crash y should be approximated by

$$C_{crash}^{AV}\left( \boldsymbol{y} \right)=C_{crash}^{car}+\Delta C_{crash}^{AV}$$

**S.E. 2**

Where y is a dummy variable vector **y** with entries equal to

$$\boldsymbol{y}=\left[ \begin{aligned} PDO \\ Minor \\ Severe \\ Fatal \end{aligned} \right]$$

To model the effect of collateral damage on accident type, we use the probability of totaling a car—i.e., having the entire cost of a car be incurred during an accident—p_total_ = 0.14. Further adjusted this probability by the four categories of severity using a probability of damage vector **d**, such that

$$\boldsymbol{d}=\left[ \begin{aligned} 0.01 \\ 0.05 \\ 0.95 \\ 0.99 \end{aligned} \right]$$

Using these definitions, we estimate the marginal cost of a crash of a given type attributable to vehicular damage via,

$$\Delta C_{crash}^{AV}\left( \boldsymbol{y},\boldsymbol{d},p_{total},C_{car},C_{AV} \right)=p_{total}\left( \boldsymbol{y\cdot d} \right)\left( \frac{C_{AV}-C_{car}}{C_{car}} \right)$$

**S.E. 3**

Combining S.E. 2 and S.E. 3 yielded plausible ranges for the cost of a accident of a given type in an AV.

**S1.** List of model variables and root values.

**S2.** Model distributions, root values, and high/low values.

**S3.** Model tables.

**S4.** Decision tree model for the baseline model: human-piloted vehicles (HPVs) versus autonomous vehicles (AVs). The model was run over N=10,000 iterations for T=100 timesteps each. The MAIS is the Maximum Abbreviated Injury Score.

**S5.** Model 2: HPV vs. AV 5-year projection

**S6.** Model 3: HPV vs. AV Taxi

**S7.** Model 4: Human-Piloted Taxi vs. AV

**S8.** Example results of Monte Carlo simulation for Model 1: HPV vs. AV (N = 10,000 microsimulations).

**S9.** Example results of Monte Carlo simulation for Model 2: HPV vs. AV 5-year projection (N = 10,000 microsimulations).

**S10.** Example results of Monte Carlo simulation for Model 3: HPV vs. AV Taxi (N = 10,000 microsimulations).

**S11.** Example results of Monte Carlo simulation for Model 4: Human-Piloted Taxi vs. AV (N = 10,000 microsimulations).

**S12.** Sensitivity analysis varying cost of AV and probability of crash in AV for Model 2: HPV vs. AV 5-year projection.
